# Hybrid *de novo* genome assembly and comparative genomics of three different isolates of *Gnomoniopsis castaneae*

**DOI:** 10.1038/s41598-023-30496-0

**Published:** 2023-02-27

**Authors:** Silvia Turco, Angelo Mazzaglia, Mounira Inas Drais, Giorgia Bastianelli, Paolo Gonthier, Andrea Vannini, Carmen Morales-Rodríguez

**Affiliations:** 1 Dipartimento di Scienze Agrarie e Forestali, Università degli Studi della Tuscia, 01100 Viterbo, Italy; 2 Dipartimento per l’innovazione nei sistemi biologici, agroalimentari e forestali, Università degli Studi della Tuscia, 01100 Viterbo, Italy; 3 Dipartimento di Scienze Agrarie, Forestali e Alimentari, Università degli Studi di Torino, 10095 Grugliasco, Italy

**Keywords:** Fungal genomics, Pathogens, Comparative genomics, Genome assembly algorithms

## Abstract

The first genome assemblies of *Gnomoniopsis castaneae* (syn. *G. smithogilvyi*), the causal agent of chestnut brown rot of kernels, shoot blight and cankers, are provided here. Specifically, the complete genome of the Italian ex-type MUT401 isolate was compared to the draft genome of a second Italian isolate (GN01) and to the ICMP 14040 isolate from New Zealand. The three genome sequences were obtained through a hybrid assembly using both short Illumina reads and long Nanopore reads, their coding sequences were annotated and compared with each other and with other Diaporthales. The information offered by the genome assembly of the three isolates represents the base of data for further application related to -omics strategies of the fungus and to develop markers for population studies at a local and global scale.

## Introduction

The fungus *Gnomoniopsis castaneae* G. Tamietti (syn. *G. smithogilvyi* L.A. Shuttleworth, E.C.Y. Liew & D.I. Guest)^1,2^, belonging to the Gnomoniaceae family, is mainly known as the cause of the “brown rot” of chestnuts, and one of the limiting factors affecting the fruit production industry. The name “brown rot” refers to the typical symptomatology, which includes a progressive rot and browning of the endosperm and embryo^1^. The disease is present in the main chestnut production areas worldwide including Australia, New Zealand, Asia, North America, South America, and Europe, affecting *Castanea sativa*, *C. mollissima* and *C. sativa* x *C. crenata* hybrids^3–7^. The fungus’s ecology and its disease cycle appear particularly complex. *Gnomoniopsis castaneae* commonly lives as endophyte or saprotroph on plant tissues and organs from which it can be easily isolated in pure culture, including leaves, buds, twigs, bark tissues, fruits and it is apparently absent from roots. Once the tree undergoes environmental stresses, *G. castaneae* may shift to a pathogenic behaviour causing bark cankers, shoot blight, leaf necrosis and fruit rot.^4,8,9^. It has also been reported as an endophyte in *Fraxinus ornus* L., *Corylus avellana* L., *Quercus ilex* L., *Q. cerris* L., and *Pinus pinaster* Aiton^6^. Beside this endophytic lifestyle, indirect evidence suggests that the colonisation of fruits occurs through infection of female flowers by external inoculum^10^, while the conditions that can cause the development of shoots and bark necrosis are still to be clarified, specifically regarding the role of the endophytic inoculum^11,12^. Epidemiologically, the impact of *G. castaneae* to chestnut is associated to specific climatic and ecological co-drivers that boost the massive production and dispersal of the inoculum^13,14^. Indeed, the outbreak of *G. castaneae* in Europe followed chronologically the invasion of the Chinese gall wasp *Dryocosmus kuriphilus* Yasumatsu, on whose galls the pathogen causes severe necrosis and abundant production of fruiting structures^8,11^. Last but not the least, the origin of the pathogen is still under debate. The synonymy with previously described fruit pathogens such as *Phomopsis castanea* and *Amphiportae castaneae* encouraged the speculation that they may be the same species^6^. Depending on the infection stage, the nut rot induced by *G. castaneae* may be confused with the one induced by *Phoma endogena*, as well as the bark cankers in young branches may be confused with the one induced by *C. parasitica*^9^. Furthermore, since living as an endophyte would be symptomless, *G. castaneae* incidence can be easily underestimated, when the diagnosis is curried out only by visual monitoring^15^. Thus, molecular analysis like MultiLocus Sequence Typing^2,9,16^, as well as SSR and HRM techniques^15,17^ have been extensively carried out for the species characterization. More recently, a specific TaqMan qPCR assay has been developed to detect and quantify the presence of the pathogen, in both symptomatic and asymptomatic tissue^18^. Nonetheless, whole genome sequencing and *de novo* assembly provide a more powerful method to support omics studies aiming to clarify different aspects of *G. castaneae* biology and lifestyle. Indeed, this acquired knowledge provides the foundation for more advanced studies regarding molecular interactions with the host driving its pathogenicity and disease expression, as well as phylogenetic analysis and genetic population studies at a global level. Thus, here we provide the first three genome assemblies of *G. castaneae*, with the ex-type MUT401 assembled at the chromosomal level, together with two additional isolates, the Italian GN01 isolate and the ICMP 14040 isolate from New Zealand, assembled at contigs level.

## Results

### DNA extraction and sequencing

Starting from 1 g of lyophilized mycelium, it was possible to extract 40 $\upmu$g (GN01), 122 $\upmu$g (MUT401) and 200 $\upmu$g (ICMP 14040) of high molecular weight (HMW) DNA from the three different samples, with the 260/230 and 260/270 nm ratios ranging from 1.7 and 2. Nonetheless, the Illumina sequencing produced high quality 2 $\times$ 5M reads (150 bp paired end), while ONT MinION runs produced $\sim$ 93 k reads (0.94 Gbp) with an N50 of 23,292 from MUT401, $\sim$ 8.9 M reads (7.9 Gbp) with an N50 of 1.512 from GN01 and $\sim$ 780k reads (4.8 Gbp) with an N50 of 14,659 from ICMP 14040, respectively, all with an average quality of 11 (Table S1).

### Genome assembly

**Figure 1:**
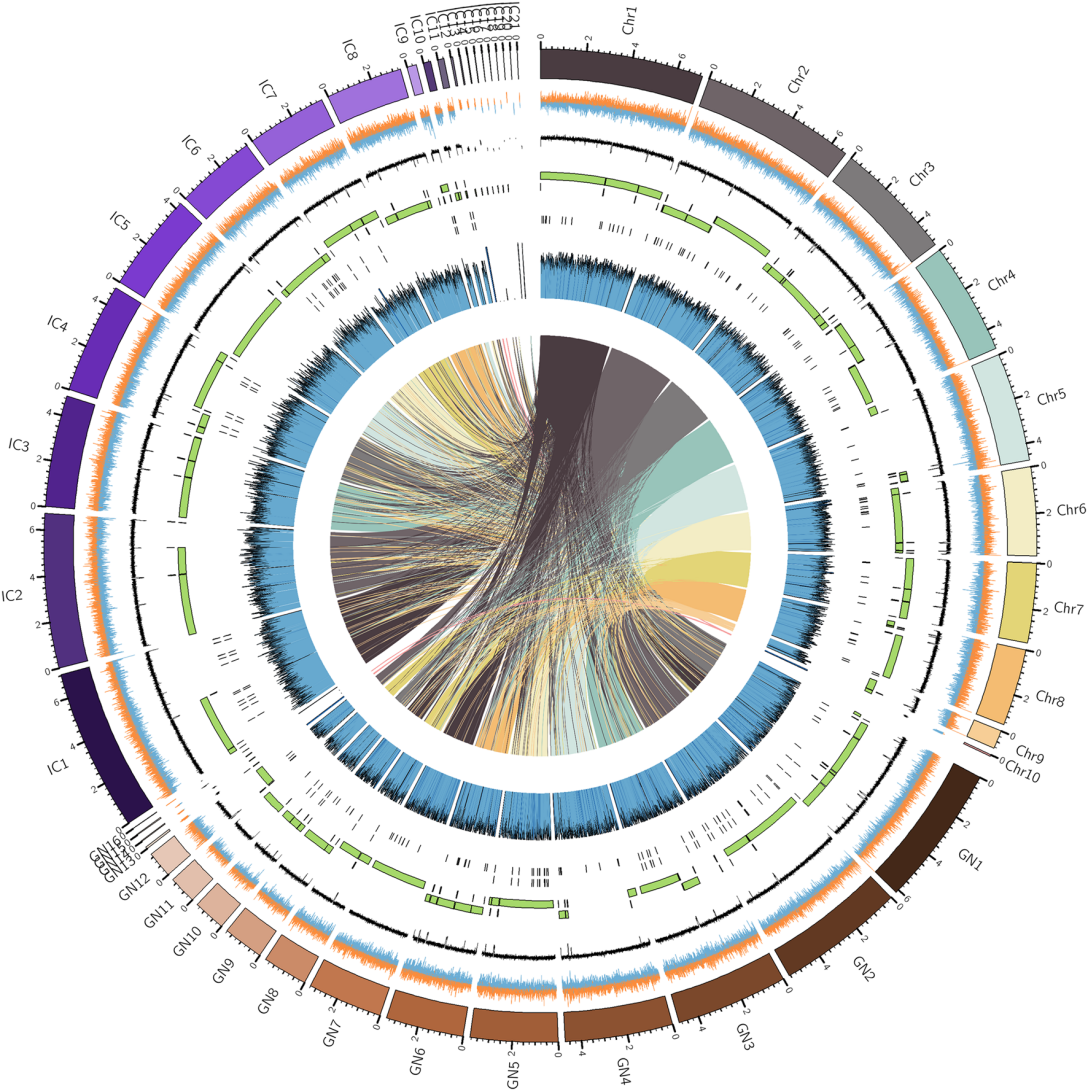
Circos plot showing the the newly assembled genomes of the three *Gnomoniopsis castaneae* isolates. Concentric circles, from outermost to innermost, show: The MUT401 complete chromosomes and the contigs related to GN01 and ICMP 14040 (indicated as GN and IC, respectively); GC skew, in orange for the forward strand and blue for the reverse strand; GC content percentage; Occultercut R1 region (in green) with an equilibrated GC content and the R0 region (in black) enriched in AT; Predicted effector proteins localized mostly in the R1 region; Illumina short reads coverage on the assembled sequences; Synteny blocks among the isolates generated by Nucmer alignment using MUT401 as reference, indicated as connected coloured links.

The reads were assembled following the different pipelines mentioned in Methods and the overall results are described by the QUAST statistics in Table S2. SPAdes assembly runs with only Illumina reads gave the most fragmented draft genomes, while Canu, MaSuRCA and Minimap2-Miniasm results were quite comparable. However, among those, the draft assembled by Canu, polished with Polca and further processed by Unicycler (shortened as CPU) was chosen for both ICMP 14040 and GN01, because of the higher N50 and N75 values and the absence of undetermined bases N, when compared with MaSuRCA and Minimap2-Miniasm assemblies, polished with Polca and further scaffolded with Unicycler (shortened as MPU and MMPU, respectively). The final drafts resulted in 16 contigs, an N50 of 4,704,111 and a short reads coverage of 42.81x for GN01, while the ICMP 14040 was assembled in 21 contigs, with an N50 of 4,830,383 and an 37.03x coverage for ICMP 14040 (Table 1, Fig. 1). In both of cases, Unicycler was able to assemble the mitochondrial circular genome of 83,752 bases in GN01 and 81,515 bases in ICMP 14040 and the overall BUSCO completeness resulted to be around 96.92% and 96%, respectively (Table 1). For the same reasons (higher N50, N75 and no N’s), the MPU draft was chosen for the ex-type isolate MUT401 (Table S2). Further manual curation allowed to reach a complete genome assembly composed of 9 chromosomes and a circular mitochondrial genome (Fig. 1). Finally, the three assemblies were deposited on the NCBI assembly database under the accession numbers JAPFGM000000000 (MUT401), JAPFGO000000000 (GN01) and JAPFGN000000000 (ICMP 14040).

**Table 1:** Assembly statistics and annotation features.

	MUT401	GN01	ICMP 14040
Reference size (bp)	40,287,698	39,394,325	39,318,709
Number of short reads	10,416,518	11,895,710	10,146,642
Number of long reads	93,417	8,924,383	780,713
Average ONT read length	10,101	890	6,197
Number of chromosomes/contigs	10	16	21
Number of chromosomes/contigs 5000	10	13	12
GC percentage	50.65 %	50.70 %	50.79 %
N50	4,890,402	4,704,111	4,830,383
N75	3,857,961	3,257,302	3,503,201
Mean coverage	38.12$\times$	42.81$\times$	37.03$\times$x
Mitochondrial genome size	78,593	83,752	81,515
BUSCO completeness	96.30%	96.92%	96%
Number of predicted coding genes	10,053	10,085	9,903

### Phylogenetics analysis and comparative genomics

**Figure 2:**
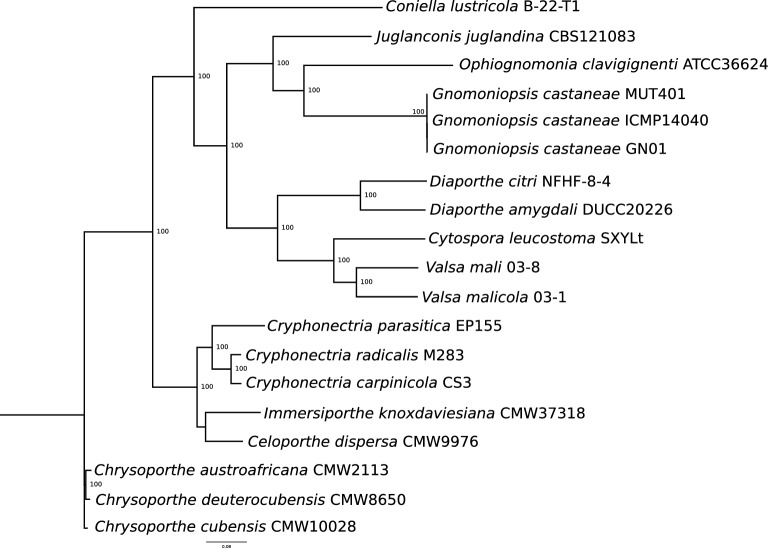
Maximum Likelihood phylogenetic tree based on the 15,041 SNPs in the core genome of the nineteen Diaporthales under comparison. The number of bootstraps is indicated as well.

**Figure 3:**
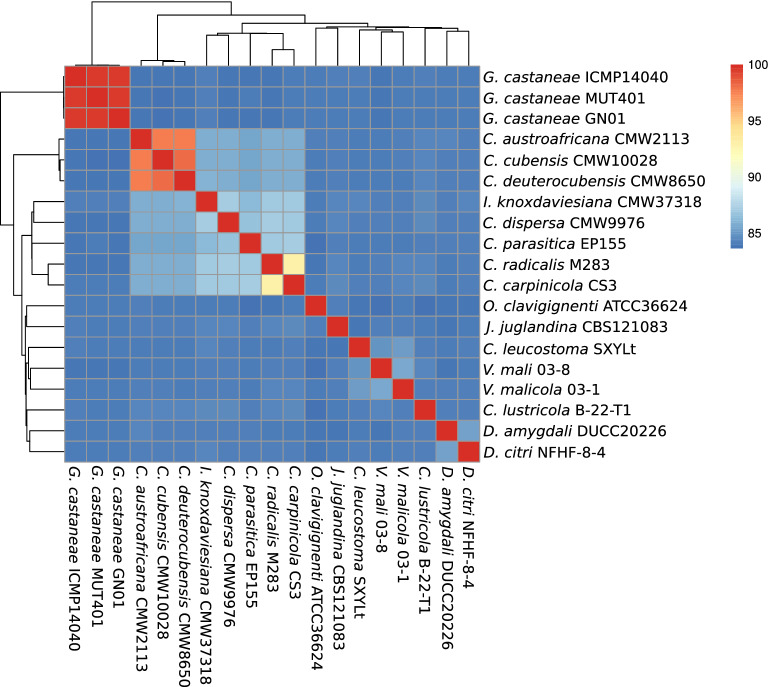
Heatmap of the Average Nucleotide Identity (ANI) analysis performed with the pyANI script and MuMMER showing the percentage of identify among the different Diaporthales.

The Maximum Likelihood (ML) phylogenetic tree built on the 15,041 SNPs on the core genome identified by Panseq among the nineteen isolates under comparison is shown in Fig. 2. The three newly assembled *G. castaneae* isolates clustered together and close to the *O. clavigignenti*, belonging to the same Gnomoniaceae family. A second ML built on the 4,332 orthogroups identified by OrthoFinder further supports the previous clusterization (Fig. S1). Furthermore, the Average Nucleotide Identity (ANI) analysis performed with the pyANI script and MuMMER and showed as an heatmap in Fig. 3, indicates a high sequence similarity between the three *G. castaneae* isolates, even if from different geographic origins, reaching the value of 99.6%. An overall percentage of identity of 83 was shared with the other Diaporthales, even with *O. clavigignenti* with which they clustered in the SNPs and species trees.

### Structural and functional annotation

**Figure 4:**
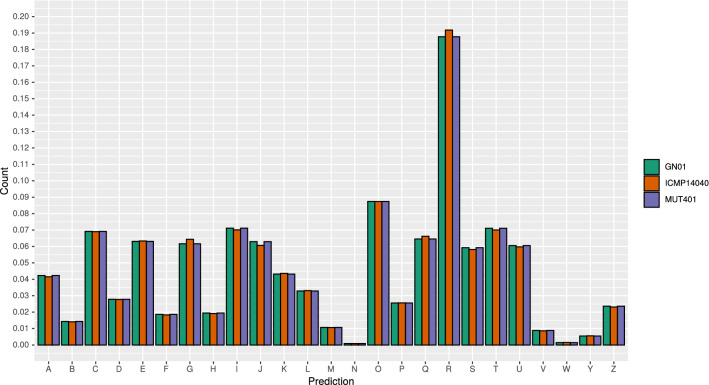
Frequency of the annotated euKaryotic Orthologous Groups (KOG). A: RNA processing and modification, B: Chromatin structure and dynamics, C: Energy production and conversion, D: Cell cycle control, cell division, chromosome partitioning, E: Amino acid transport and metabolism, F: Nucleotide transport and metabolism, G: Carbohydrate transport and metabolism, H: Coenzyme transport and metabolism, I: Lipid transport and metabolism, J: Translation, ribosomal structure and biogenesis, K: Transcription, L: Replication, recombination and repair, M: Cell wall/membrane/envelope biogenesis, N: Cell motility, O: Posttranslational modification, protein turnover, chaperones, P: Inorganic ion transport and metabolism, Q: Secondary metabolites biosynthesis, transport and catabolism, R: General function prediction only, S: Function unknown, T: Signal transduction mechanisms, U: Intracellular trafficking, secretion, and vesicular transport, V: Defense mechanisms W: Extracellular structures, Y: Nuclear structure, Z: Cytoskeleton.

A total of 10,053 putative genes, 142 tRNAs and 46 rRNAs were identified in MUT401, 10,085 putative genes, 147 tRNAs and 35 rRNAs were identified in GN01, while 9,903 putative genes, 150 tRNAs and 47 rRNAs were annotated in ICMP 14040. Among these, TargetP identified 1,134 proteins with a signal peptide, 339 with a mitochondrial transit peptide in MUT401, 1,113 proteins with a signal peptide, 340 of which with a mitochondrial transit peptide in GN01, while ICMP 14040 showed 1,056 signals and 334 mitochondrial transit peptide (Fig. S2). SignalP was also able to recognize most of the signal peptides coming from all the three isolates as the standard secretory signal peptides transported by the Sec translocon and cleaved by Signal Peptidase I. KEGG annotations were quite comparable among the three isolates, with the most abundant categories represented by the enzymes involved in metabolism, followed by membrane trafficking and chromosomes and associated proteins from the Genetic information processing cluster (Fig. S3). Same results were obtained by KOG annotations, with the class categories O (Post-translation modification, proteins turnover and chaperons), T (signal transduction mechanisms), I (lipid transport and metabolism) and C (energy production and conversion) being the most abundant, after the uncharacterised R class of general function prediction only (Fig. 4). The alternapyrone biosynthetic gene cluster belonging to the T1PKSs was identified by antiSMASH6 in both MUT401 and GN01 with 100% of similarity and in ICMP 14040 with 80% of similarity. Among the other clusters identified, it is worth to mention the terpene Squalestatin S1 ($\sim$40% of similarity), the NRPS cyclochlorotine (37%) and naphthalene (33%), T1PKS betaenone A,B and C (37%) and the wortmanamide A and B (33%)(Table S3).

**Figure 5:**
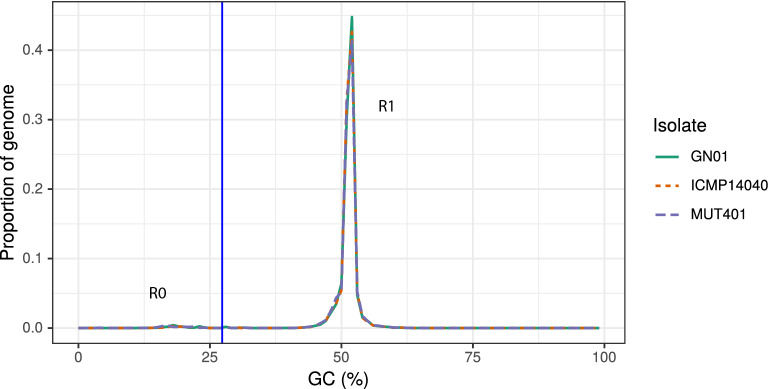
Bimodal distribution of the GC content in the three genomes identified identified by Occultercut as enriched in AT (R0) and GC equilibrated (R1) using a GC content threshold indicated by the vertical blue line.

### Repeated sequences and AT-rich regions

Based on the REPET pipeline analysis, the three isolates resulted in a relatively low abundance of repetitive elements. In particular, the MUT401 isolate contains 19 repetitive consensus sequences, covering 839,120 bp (2.08% of the whole genome). Of these, seven sequences were retrotransposons of Class I (2 LARD, 2 LTR Copia and 3 LTR Gypsy), 3 were DNA-intermediate transposons of Class II (1 MITE and 2 TIR), 8 unclassified and 1 confused among Class I LTR and DIRS. In GN01, instead, 15 total consensus sequences were identified, covering a total region of 535,564 bp, representing the 1.36% of the whole genome: five sequences are related to Class I (1 LARD, 2 LTR Copia and LTR Gypsy), 4 to Class II (1 MITE and 3 TIR), and six sequence resulted to be unclassified. Interestingly, ICMP 14040 contains only 10 consensus repetitive sequences, covering 38,323 bp of the genome (only 0.09% of the whole genome): 4 sequences belong to Class I (1 LARD, 2 LTR Copia and 1 LTR Gypsy), with 5 unclassified and 1 confused. These results were quite in line with those deriving from RepeatModeler and RepeatMasking embedded within the MAKER annotation pipeline, giving 16, 11 and 15 repetitive elements in MUT401, GN01 and ICMP 14040, respectively. The surveyed genomes presented a bimodal GC-content distribution when analysed with OcculterCut, with two peaks corresponding to the R0 AT-rich region and to the R1 GC-equilibrated region, in the GC-content distribution plot (Fig. 5). In particular, the R0 with an AT-rich peak represented the 1.96% of the MUT401 genome with an average GC% of 17.6, the 1.91% of GN01 with an average GC% of 18.3 and only the 1.53% of ICMP 14040 with an average GC content of 18.8. Furthermore, the frequencies of the 16 possible di-nucletotides were calculated with OcculterCut in order to evaluate the possible RIP (Repeat Induced Point mutation) involvement in the AT-rich region formation. As expected, the frequencies of AA, AT, TT and TA were higher in the AT-rich region, with the latter slightly higher over the others (Fig. S4). This result, together with the RIP ratio values (TpA/ApT > 1.50 and (CpA + TpG)/(ApC + GpT) < 0.5) may indicate RIP activities in the three isolates.

### Pathogenicity related features

**Figure 6:**
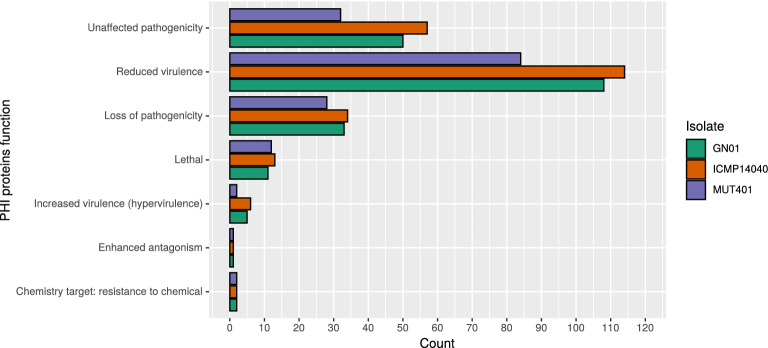
Putative pathogenicity-related features identified by BLASTp alignment towards the PHI database.

Among the putative annotated genes, a total of 417 (MUT401), 396 (GN01) and 424 (ICMP 14040) carbohydrate-active enzymes (CAZymes) were detected, the majority of which encoding intracellular glycoside hydrolases (Ghs), followed by intracellular glycosyltransferases (Gts) and auxiliary activity (AAs) (Fig. S5). Furthermore, by BLASTp alignment of the annotated proteins towards the PHI database, 151 genes involved in pathogenicity were identified in MUT401, 150 genes in GN01 and 166 genes in ICMP 14040, which functions is defined according to the high-level phenotype outcomes used in PHI-base (Fig. 6)^19^.

## Discussion

The present study reports the first complete genome sequences of three different isolates of *G. castaneae*: the Italian ex-type MUT401, a second Italian GN01 isolate from the Viterbo area and the ICMP 14040 isolate from New Zealand. To reach these results, a hybrid assembly approach was chosen, in order to combine the advantages of the Illumina short reads sequencing method, providing high depth coverage, and of the Oxford Nanopore long reads (ONT) sequencing technology, providing longer scaffolds. Indeed, these two methodologies have been successfully used in several fungal genome assembly, even in more complex situations with genome enriched in transposon and tandem repeats^20,21^. As an example, using the hybrid assembly approach, complete, near complete or draft genomes have been assembled in *Pyricularia oryzae* (syn. *Magnaporthe oryzae*)^22^, *Aspergillus flavus*^23^, *Ceriporia lacerata*^24^ and *Fusarium* sp.^25^.

The hybrid assembly efficiency depends on both the quality and quantity of the starting material, and thus, the choice of the DNA extraction method is important. In fact, for Third Generation Sequencing (TGS) technologies such as Oxford Nanopore or PacBio sequencing, the use of column-based methods is discouraged because of the resulting highly fragmented DNA, while the beads-based methods or the manual protocols based on CTAB followed by phenol-chloroform extraction are preferable because they better preserve the DNA integrity. Nonetheless, the fungal species and their growth conditions may influence the final extracted DNA yield and, in this regard, *G. castaneae* resulted very recalcitrant. For this reason, it was necessary to optimise the DNA extraction protocol proposed by Darma and McDonald (2019)^26^. In particular, a higher amount of lyophilized mycelium as starting material was used, together with higher incubation temperature for proteinase K and RNAse A, longer centrifuge times and without phenol. Following these precautions, we were able to isolate from 40 to 200 $\upmu$g of DNA, starting from 1 g of lyophilized mycelium. Based on the completeness of the assembled genomes, less but longer reads (MUT401) performed better than more but shorter reads (GN01). Indeed, starting from 1 Gb of raw data consisting of only 93K reads but with an average read length of 10K, we were able to assemble 9 complete chromosomes, from telomere to telomere, plus a circular mitochondrial genome. On the contrary, with higher amounts of reads but of shorter length, like in the case of GN01 or ICMP 14040, the genomes could be assembled only as draft, even if consisting in few contigs (16 and 21, respectively). The reasons rely on the fact that several AT rich regions of at least 4-5 K in length are spread along the genomes of the three isolates and thus, with shorter reads it was impossible for the *de novo* assembly tools to perfectly cover and assemble that region. MUT401 longer reads overcame this problem, reaching the flanking region of the AT island and filling the gap left by the GN01 and ICMP 14040 shorter reads.

By comparing the different assembly pipelines performance, as expected, the hybrid assembly performed better than the single short-reads assembly obtained with SPAdes, with Canu, MaSuRCA and Minimap-Miniasm giving comparable results in terms of number of contigs. The drafts were further aligned to each other and visualised with IGV showing really high consistency in terms of sequence segmentation. Nonetheless, MaSuRCA assembly was chosen for MUT401, whereas Canu drafts were chosen for GN01 and ICMP 14040. By giving these drafts as trusted contigs to Unicycler, it was feasible to obtain the three mitochondrial circular genomes of comparable sizes. This additional step with Unicycler to close the mitochondrial genome was previously applied in the complete genome assembly of the entomopathogenic fungus *Metarhizium brunneum*^27^. The consistency of the chosen draft (either MaSuRCA or Canu) was further verified by alignment to the other draft assembly as well as by ONT and Illumina reads alignment, followed by alignment visualisation and coverage evaluation. To reach the complete assembly of MUT401, further steps were required, starting from six chromosomes and one mitochondrion already completely assembled by MaSuRCA and Unicycler. First, raw reads containing the telomere sequences TTAGGG at their terminal ends were extracted from both ONT and Illumina datasets using a simple grep command and further aligned to the MPU draft in order to identify those contigs already reaching the telomeres regions. These reads were aligned and manually compared to those contigs without telomeres, to finally fill all the gaps and reach nine telomere to telomere chromosomes, supported by an average coverage of 38.12x.

When aligned to each other, the three isolates showed a genetic identity above 99.6%, suggesting a really high conserved genome, despite the different geographic origins and the AT-rich isochores spread among the genome. These AT-rich region may derive from Repeat-Induced Point mutation (RIP) events, operated by the product of the cytosine methyltransferase homologue (rid) gene. This mechanism is used as a protection against the transposable elements, by cytosine methylation which is then mutated into thymine^28–30^. Margolin et al.^31^ introduced the TpA/ApT and (CpA+TpG)/(ApC+GpT) ratios to assess the likelihood that regions have been mutated by RIP. In general, TpA/ApT > 1.50 and (CpA + TpG)/(ApC + GpT) < 0.5, as the ones calculated by RIPCAL for the three *G. castaneae* isolates, may indicate a RIP event. Despite the relatively low abundance of AT-rich regions and the dinucletotides ratios, which suggest the possibility of Repeat Induced Point mutation (RIP) events, no RIP-defective (rid) DNA methyltransferase gene similar to those investigated by Freitag et al.^32^ was found in the genome of the three isolates and all the annotated genes ended into the R1 region with an equilibrated GC content.

In both phylogenetics analysis, *G. castaneae* isolates clustered within the same clade of *O. clavigignenti*, the only other representant of the Gnomoniaceae family included in the tree . However, when aligning the whole genome sequence in the Average Nucleotide Identity (ANI), the three *G. castaneae* isolates clustered far away from *O. clavigignenti*. This may be explained by the fact that, by being based on the whole genome similarity, the ANI analysis includes the accessory genome as well, which provides an important contribution to the genetic diversity and plasticity, even among closely related strains. Regarding plasticity, among the Diaporthales genomes available on NCBI, only four genomes are completely assembled in 13 or 14 chromosomes, with a gene content ranging from 9k to 15k genes, in line with our findings. When more complete genomes will be available, further studies to identify the accessory chromosomes of these pathogens could be carried out^30,33^. The overall structural and functional annotations resulted to be comparable as well, especially in the number of codifying genes, KEGG, KOG annotations and CAZymes. Not surprisingly, the ex-type MUT401 isolate, resulted to have less Plant-Host interaction related proteins (of the classes unaffected pathogenicity, reduced virulence and loss of pathogenicity) when compared with the other two isolates and the effectors proteins are all located outside the AT-rich regions identified by Occultercut.

Thanks to the availability of the *Gnomoniopsis castaneae* genome, new genetic studies regarding the origin of the pathogen, the epidemiology of chestnut brown rot and the interactions of the fungus with its host will be now possible. Indeed, these studies will allow the development of effective control strategies against this emerging and economically important pathogen.

## Methods

### Fungal material

Three *Gnomoniopsis castaneae* cultures were used in the present study: the ex-type MUT 401 isolated from fruit in 2007 in Northern Italy^2^; the GN01 isolated from diseased fruit in 2019 in Central Italy and stored in the collection of the Department for Innovation in Biological, Agro-food and Forest systems (DIBAF); the ICMP 14040 from the International Collection of Microorganisms from Plants (ICMP - New Zealand) isolated from healthy leaves of *Castanea* sp. in 1999 in New Zealand^34^. Single hyphae subcultures of the three isolates were grown on Potato Dextrose Agar (PDA) (Oxoid, Basingstoke, UK, 39 $g \cdot l^{-1}$) at $20\pm 1^{\circ }$C. To obtain abundant mycelium, a plug of each isolate was plated on 15 mL of Potato Dextrose Broth (PDB) (MP Biomedicals, Irvine, USA) in 90mm Petri dishes. After 7 days of incubation in the dark at $20\pm 1^{\circ }$C, mycelium was collected and dried on sterile paper under the flow chamber. Mycelium was frozen at -$80^{\circ }$ C and lyophilized (Edwards Modulyo Freez-Drier, England) at -48–$-55^{\circ }$C for 8 h.

### High molecular weight DNA extraction

DNA was extracted following the protocol of Darma and McDonald (2019)^26^, with some modifications. Briefly, lyophilized mycelium was grinded with mortar and pestle in liquid nitrogen. The fine powder was then transferred into a 50 mL falcon and incubated for 45 minutes at $60^{\circ }$C with 16 mL of lysis buffer (10% CTAB, 0.5 M EDTA, 1 M Tris-HCl pH 8, 4 M NaCl) and 200 $\mu$l of proteinase K (20 mg/ml). One volume of chloroform:isoamyl alcohol (24:1) was then added to the solution, mixed slowly and centrifuged at 5,000 g for 20 min at room temperature. The aqueous phase was then incubated with 30 $\mu$l of RNAseA (10 mg/ml) and further extracted with a second round of chloroform:isoamyl alcohol. The aqueous phase was incubated with the precipitation buffer (10% CTAB, 0.5 M EDTA, 1 M Tris-HCl pH 8) for 30 min at $55^{\circ }$C under constant agitation and then centrifuged for 40 min at 7,500 g. The pellet was then washed twice with 70% ethanol and finally dissolved in 400 $\mu$l of TRIS (pH 8). The DNA was quantified using the Invitrogen Qubit fluorometer (Thermo Fisher Scientific, Massachusetts) while the purity and ratios were evaluated in a 0.5% agarose gel electrophoresis run and with Thermo Scientific Multiskan GO (Thermo Fischer Scientific,Massachusetts), respectively.

### Library preparation and genome sequencing

For Oxford Nanopore Technologies (ONT, United Kingdom) sequencing, the DNA was first prepared end-repaired using NEBNext Ultra II End Repair/dA Tailing Module (New England BioLabs) and the libraries were prepared with the Ligation sequencing kit (SQK-LSK109) and the Native Barcoding Expansion 1-12 (EXP-NBD104). Two different sequencing runs were performed on a MinION Mk1C device (ONT, United Kingdom) using two R9.4.1 flow-cells (ONT). The basecalling of the ONT long reads was performed using Guppy within the MK1C device and the reads quality and sequencing statistics were evaluated using NanoPlot v.1.30.1^35^. An aliquot of the same DNA sample was sequenced at Eurofins Genomics (Eurofins Genomics GmbH, Konstanz, Germany) with the genome sequencer Illumina NovaSeq 6000 S2 using the paired-end sequencing. The quality of the paired-end Illumina reads was evaluated using FastQC^36^, before downstream analysis.

### *De novo* hybrid assembly

To compare their performance and to obtain the best quality genomes, four different assembly tools were tested in parallel. SPAdes v3.11.1 was used with Illumina short reads assembly only^37^ while Canu v2.1.1^38^, MaSuRCA v3.4.2^39^ and Minimap2 v2.12-r849-dirty^40^ in combination with Miniasm v0.3-r179^41^, were used in separated run for hybrid assembly using both Illumina and ONT reads. All of them were used with default parameters, setting an expected genome size at 40 Mb in Canu. Each assembly was further polished by Polca^42^ and used as trusted contig reference for a further assembly step with Unicycler v0.4.9b Wick et al.^43^ to get the closed circular mitochondrial genome. The assembly quality statistics were evaluated using QUAST v5.0.2^44^, while the assembly completeness was evaluated with BUSCO v5.beta.1^45^, using Sordariomycetes_db10 as ortholog lineage dataset which consists in a set of 3,817 conserved profiles. For each isolate, the draft genomes deriving from the different tools were compared to each other through BWA v0.7.12 alignment^46^ and by manual inspection through the Integrative Genomic Viewer (IGV). Finally, one assembly per isolate was chosen for downstream analysis.

### Genome annotation

The *de novo* MAKER pipeline v3.01.03^47^ was used to structurally annotate the assembled genomes. Within the pipeline, the built-in RepeatModeler was used to mask repetitive elements, SNAP and AUGUSTUS were used for an *ab initio* gene prediction, Est2Genome and Protein2Genome were used to refine introns and exons boundaries, while Exonerate and tRNAscan-SE were used to identify the genes related to the tRNA biosynthesis. Among the Diaporthales genomes that were available on the NCBI database at the time of the analysis, sixteen isolates, one per each species, have been selected for phylogenetics and comparative genomic analysis (Table 2). Only six genomes were already annotated and thus, their transcripts and proteins were concatenated into two separated files and used as prediction models within the MAKER pipeline. The remaining ten genomes were annotated here following the same MAKER pipeline described above for the three *G. castaneae* isolates.

**Table 2:** List of Diaporthales isolates used for phylogenetics and comparative analysis.

Organisms species	Isolate	Accession number
*Celoporthe dispersa*	CMW9976	GCA_016584495.1
*Chrysoporthe austroafricana*	CMW2113	GCA_001051155.2
*Chrysoporthe cubensis*	CMW10028	GCA_001282315.2
*Chrysoporthe deuterocubensis*	CMW8650	GCA_001513825.2
Coniella lustricola*	B22-T-1	GCA_003019895.1
*Cryphonectria carpinicola*	CS3	GCA_014849955.1
Cryphonectria parasitica*	EP155	GCF_011745365.1
*Cryphonectria radicalis*	M283	GCA_014849355.1
Cytospora leucostoma*	SXYLt	GCA_003795295.1
*Diaporthe amygdali*	DUCC20226	GCA_021655905.1
Diaporthe citri*	NFHF-8-4	GCF_014595645.1
*Immersiporthe knoxdaviesiana*	CMW 37318	GCA_021117315.1
*Juglanconis juglandina*	CBS121083	GCA_003012975.1
*Ophiognomonia clavigignenti-juglandacearum*	ATCC36624	GCA_003671545.1
Valsa mali*	03-8	GCA_000818155.1
Valsa malicola*	03-1	GCA_003795315.1

The annotation of the isolates indicated by an asterisk were already available on NCBI and were here used as models for the gene predictions.

### Phylogenetics analysis based on whole genome and proteins comparison

Panseq^48^ was used to identify the core genomes among the 19 isolates under comparison and a Maximum likelihood (ML) tree was built on the identified SNPs using raxmlHPC with GTRCATI algorithm as substitution model and 1,000 bootstraps^49^. Orthologous proteins were identified using OrthoFinder and the results were used to build a species tree^50^. Both trees were visualised in a dendrogram using FigTree v1.4.41^51^. The Average Nucleotide Identity (ANI) among the different isolates was calculated with the pyani script, which includes the MUMMer algorithm^52^.

### Repetitive elements analysis

For a *de novo* identification of repetitive elements, both REPET pipeline v2.2^53^ and RepeatModeler (which includes RepBase for TE classification and RepeatMasker for repeats masking) within the MAKER pipeline were used^54,55^. OcculterCut was used to identify AT-rich regions and their relationship to genes possibly involved in the plant-pathogens interaction, as well as possible RIP events involved in the AT-rich formation^28^. The RIP ratio values were calculated with RIPCAL^56^.

### Functional genomics

BlastKOALA for KEGG orthology was used for deeper functional annotation^57^, while biosynthetic gene clusters (BGCs) were automatically searched and analysed by AntiSMASH v6.0^58^. The carbohydrate active enzymes (CAZymes) involved in carbohydrate metabolism were identified through the dbCAN2 meta web server^59^, which includes SignalP v4.0^60^ for putative secreted proteins identification. Prediction of transmembrane proteins was performed with DeepTMTHMM^61^, while the Pathogen Host Interactions (PHI) database was used to identify pathogenicity and virulence related genes^19^.

## Supplementary Information

Supplementary Information 1.

Supplementary Information 2.

Supplementary Information 3.

Supplementary Information 4.

## Data Availability

The genomes have been deposted on the NCBI Genome database under the following Accession numbers JAPFGM000000000, JAPFGO000000000 and JAPFGN000000000. All the raw data associated with this work are available under request to the corresponding authors.
