# Supplementary material for: Hybrid de novo genome assembly and comparative genomics of three different isolates of Gnomoniopsis castaneae
Source: Sci Rep. 2023 Feb 27;13:3356. doi: 10.1038/s41598-023-30496-0 (PMC9971261; doi:10.1038/s41598-023-30496-0)
Supplement: Supplementary file 1 — Supplementary Information 1. [file 41598_2023_30496_MOESM1_ESM.pdf]

Supplementary Figures

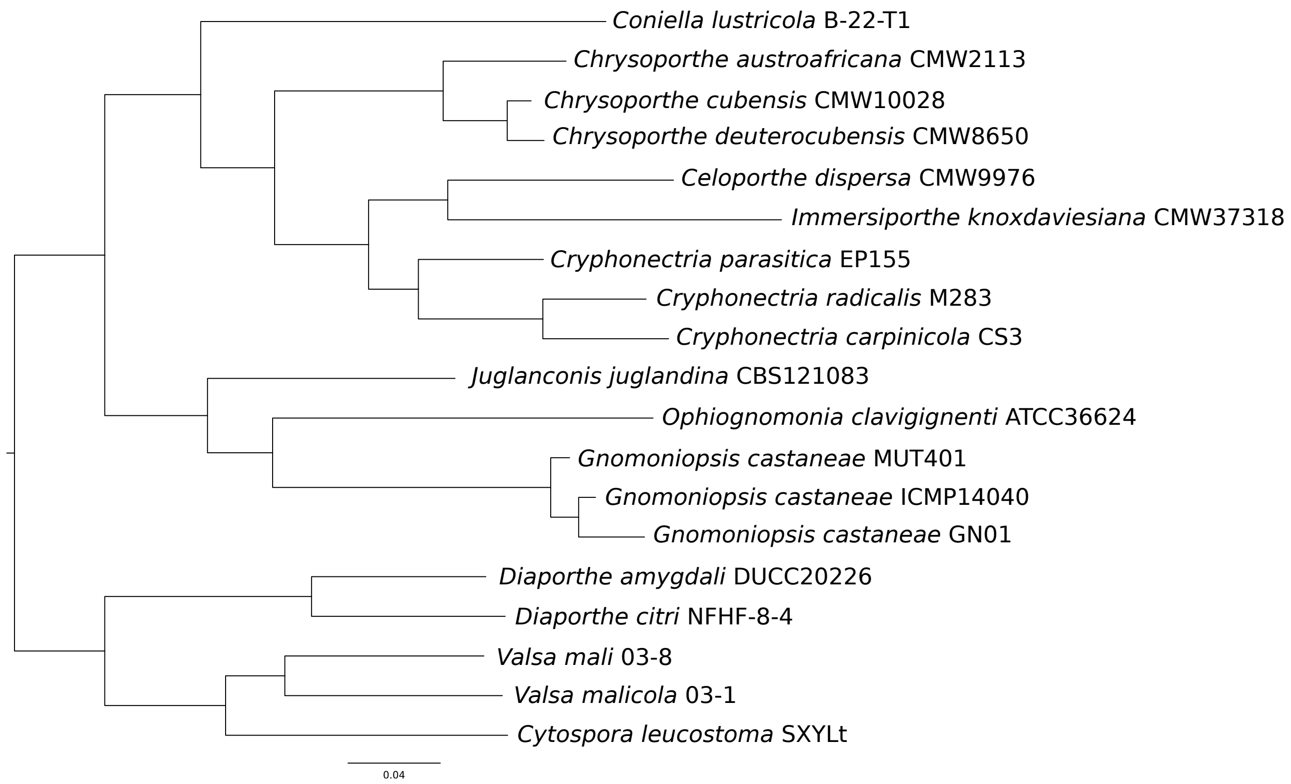

Figure 1. Maximum Likelihood phylogenetic tree of the orthologous proteins identified using OrthoFinder.
.

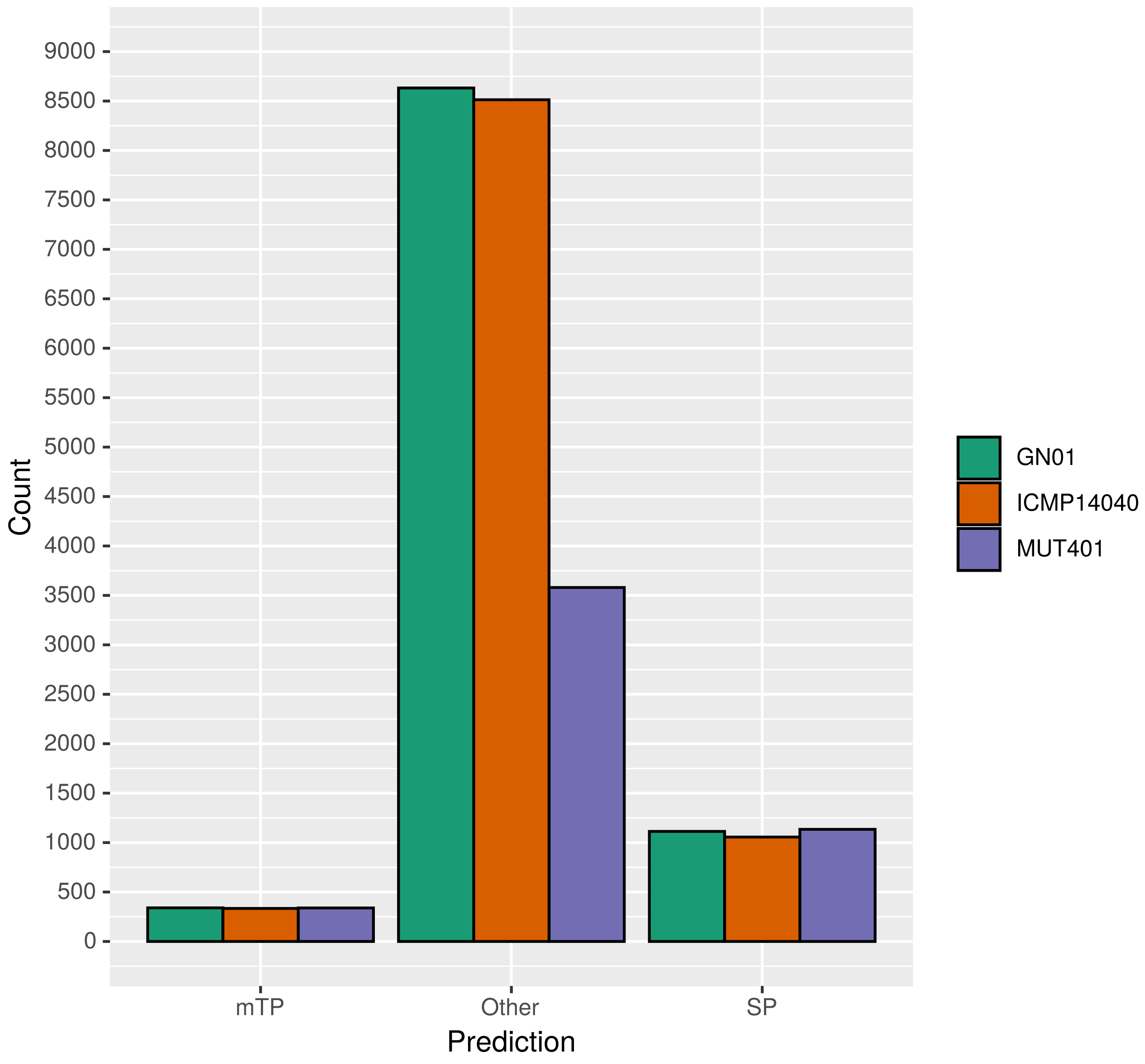

Figure 2. TargetP results showing the cumulative count of predicted proteins, among the three isolates, containing a signal peptide (SP), mitochondrial translocation signal (mTP) or other possible signal (chloroplast, extracellular, and "other" localization)

.

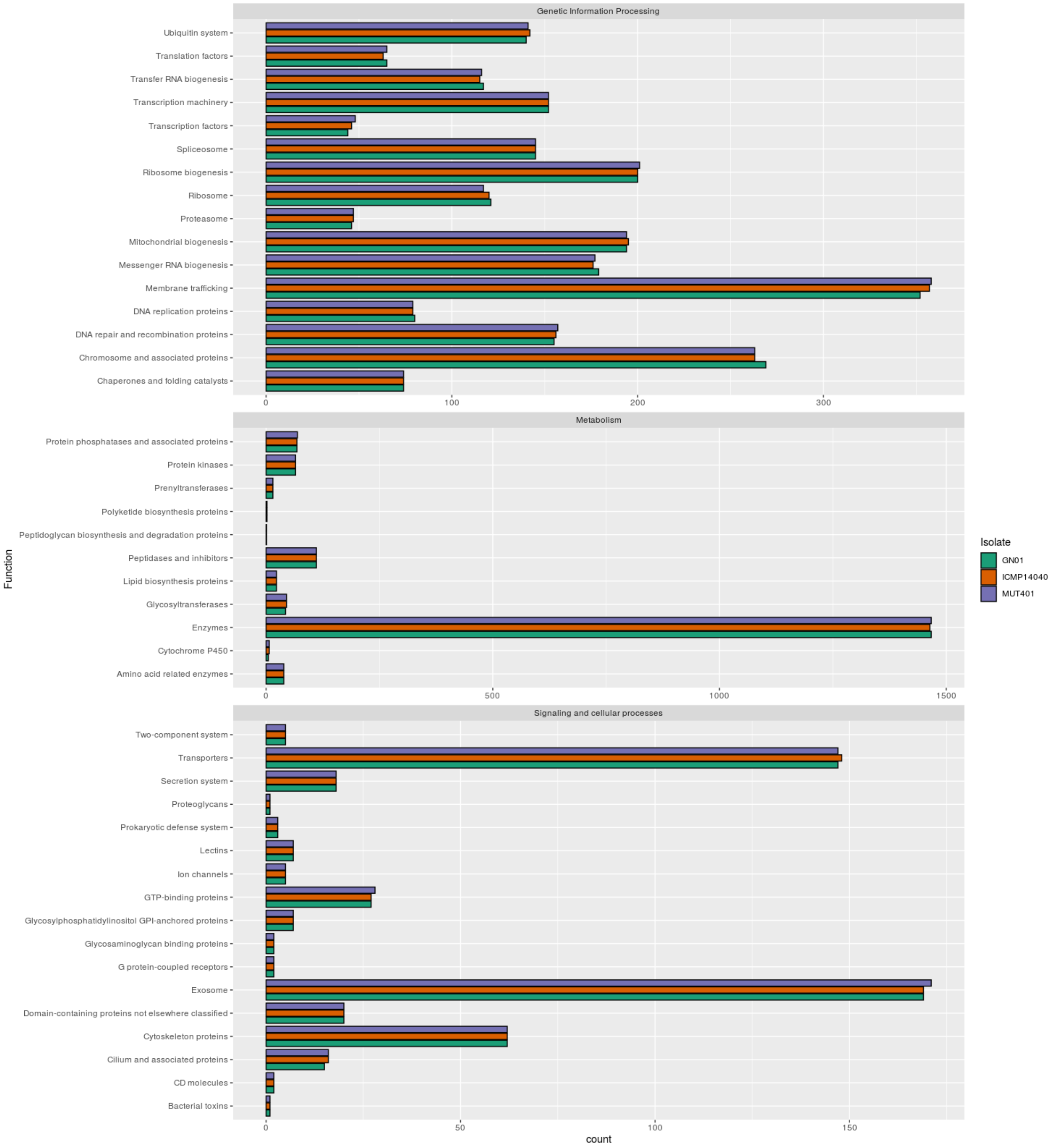

Figure 3. KEGG annotation and distribution among the different tree main classes: Genetic Information Processing, Metabolism and Signaling and Cellular Processes

.

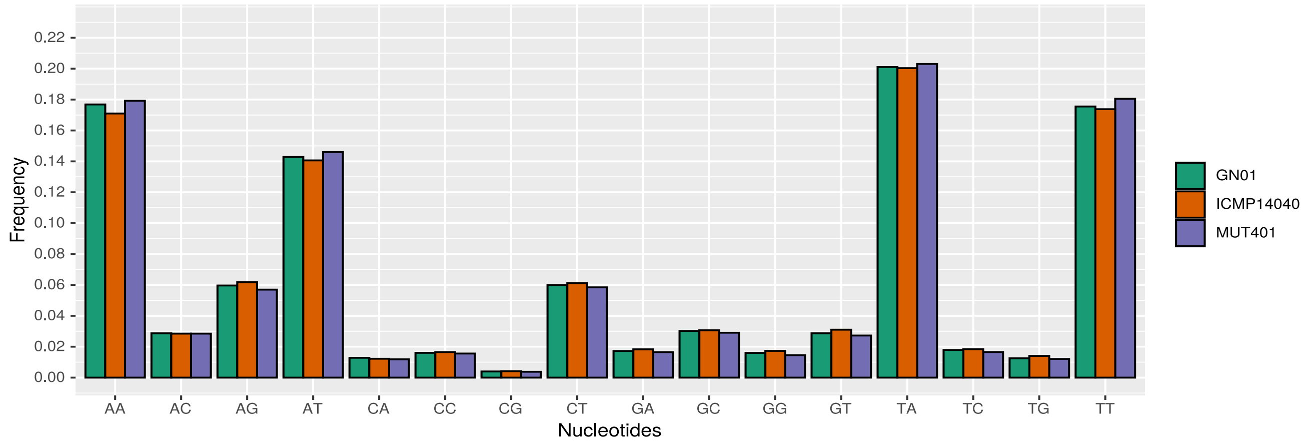

Figure 4. Dinucleotides frequencies calculated by Occultercut in the R0 region

.

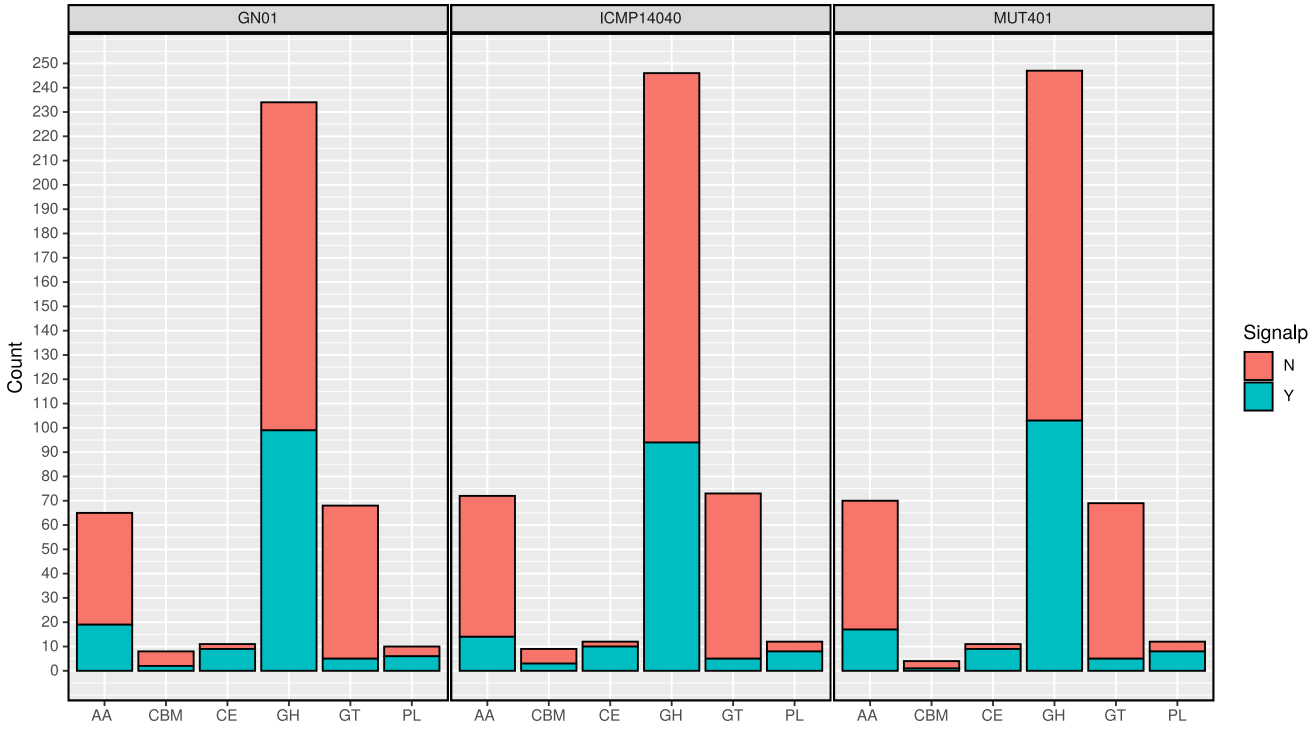

Figure 5. Count of the CAZymes families in the annoted features, with (Y) or without (N) signal peptides. AA: Auxiliary Activities, CBM: CAZymes with carbohydrate-binding modules, CE: carbohydrate esterases, GH: glycoside hydrolases, GT: glycosyltransferases, PL: polysaccharide lyases.

.
